# Brain Metabolism Alterations in Type 2 Diabetes: What Did We Learn From Diet-Induced Diabetes Models?

**DOI:** 10.3389/fnins.2020.00229

**Published:** 2020-03-20

**Authors:** Alba M. Garcia-Serrano, João M. N. Duarte

**Affiliations:** ^1^Department of Experimental Medical Science, Faculty of Medicine, Lund University, Lund, Sweden; ^2^Wallenberg Centre for Molecular Medicine, Faculty of Medicine, Lund University, Lund, Sweden

**Keywords:** diet-induced obesity, brain metabolism, insulin resistance, glucose, sucrose, high-fat

## Abstract

Type 2 diabetes (T2D) is a metabolic disease with impact on brain function through mechanisms that include glucose toxicity, vascular damage and blood–brain barrier (BBB) impairments, mitochondrial dysfunction, oxidative stress, brain insulin resistance, synaptic failure, neuroinflammation, and gliosis. Rodent models have been developed for investigating T2D, and have contributed to our understanding of mechanisms involved in T2D-induced brain dysfunction. Namely, mice or rats exposed to diabetogenic diets that are rich in fat and/or sugar have been widely used since they develop memory impairment, especially in tasks that depend on hippocampal processing. Here we summarize main findings on brain energy metabolism alterations underlying dysfunction of neuronal and glial cells promoted by diet-induced metabolic syndrome that progresses to a T2D phenotype.

## Introduction

Diabetes mellitus is among the top 10 causes of death in the world. Insulin-resistant diabetes (or T2D) often progresses from obesity, a pandemic that is favored by a sedentary lifestyle and the widespread consumption of food products rich in saturated fat and refined carbohydrates (Swinburn et al., 2011). Many factors of the metabolic syndrome impact brain function, such as chronic hyperglycemia, microvascular complications, insulin resistance, dyslipidemia, and hypertension (Duarte, 2015; Gaspar et al., 2016). There is also a growing body of epidemiological evidence suggesting that insulin resistance is associated with increased risk of developing age-related cognitive decline, mild cognitive impairment (MCI), vascular dementia, and Alzheimer’s disease (AD) (Frisardi et al., 2010; Spauwen et al., 2013; de la Monte, 2017). Brain insulin signaling deficits have been proposed to impact the brain through mechanisms that include the modulation of energy metabolism, synaptic plasticity, learning and memory, as well as interacting with Aβ and tau, the building blocks of amyloid plaques and neurofibrillary tangles (Craft et al., 1998; Steen et al., 2005; Zhao and Townsend, 2009). In addition, a plethora of studies in rodent models of diabetes suggest that both glucose neurotoxicity and deficient insulin signaling impair brain structure and function leading to behavioral and cognitive alterations (e.g., Duarte et al., 2012a, 2019; Calvo-Ochoa et al., 2014; Girault et al., 2019; Lizarbe et al., 2019b).

Hyper-caloric feeding is able to trigger insulin resistance, and diet is also a modulator of brain function and neurodegeneration. In cognitively normal individuals without obesity or diabetes, nutritional patterns were found to associate with ^11^C-Pittsburgh compound-B (marker of β-amyloid plaques) and [^18^F]-fluorodeoxyglucose (FDG; marker of glucose metabolism) accumulation (Berti et al., 2015). Berti et al. verified that an AD-protective diet includes high intake of fresh fruit and vegetables, whole grains, fish and low-fat dairies, and low consumption of sugar, high-fat food products, and processed meat. Sugar intake was also found positively associated with cerebral amyloid burden measured by [^18^F]-florbetapir positron emission tomography (PET), and negatively correlated with cognitive performance in cognitively normal subjects (Taylor et al., 2017).

Dietary imbalances may trigger metabolic disorders and obesity, which in the long-term may progress to insulin resistance and T2D. Clinical studies set obesity and associated metabolic derangements as important risk factors for dementia (Pedditzi et al., 2016; Singh-Manoux et al., 2018; Bandosz et al., 2020). Li et al. (2017) observed that cortical Aβ deposition by [^18^F]-florbetapir PET was decreased in T2D patients while Aβ levels increased in the cerebral spinal fluid. Insulin resistance is associated with AD markers, such as accumulation of ^11^C-Pittsburgh compound-B and FDG in PET scans (Willette et al., 2015a, b), or Tau-protein levels in the cerebral spinal fluid (Laws et al., 2017). Takenoshita et al. (2019) investigated AD markers, namely, amyloid and Tau protein deposition by PET, in AD associated to T2D, and concluded that there are patient subgroups with neuronal damage independent of AD pathology. It is apparent that a diabetes-related dementia can be considered a different entity from AD itself.

Although several mechanisms underpin brain dysfunction that leads to poor cognitive performance in T2D (Gaspar et al., 2016), confusion and controversy landed in the field due to the variety of phenotypes generated in experimental animal models of T2D. Nevertheless, our knowledge on brain dysfunction mechanisms upon exposure to diabetogenic diets is increasing, and may help preventing cognitive deterioration associated to poor life styles.

## Memory Dysfunction Induced by Diabetogenic Diets

Many studies in T2D animal models have employed diets rich in sugar and/or fat in order to induce T2D, namely, high-fat diet (HFD), high-sucrose diet, high-fructose diet, or the combination of some of them. Glucose intolerance develops promptly in rodents exposed to HFD, followed by a progressive increase of fasting insulin levels and metabolic derangements such as hepatic lipid accumulation (Soares et al., 2018). We have also recently reported that increasing the dietary amount of lard-based fat from 10 to 45 or 60% leads to slightly different diabetic phenotypes: compared to controls that were exposed to the low-fat diet, increased fed glycemia and plasma corticosterone were observed in mice fed a 60%- but not 45%-fat diet (Lizarbe et al., 2019b).

Similar degree of insulin resistance and of stress biomarkers in liver and pancreas have been observed in rats exposed to HFD, high-fructose diet, or the combination of both, compared to control diet (Balakumar et al., 2016). In mice, HFD feeding was found to cause elevated basal insulin levels, which was not observed in mice fed a combined high-fat and high-sucrose diet, despite similar energy intake and degree of glucose intolerance (Omar et al., 2012). The authors attributed this difference to the distinct effect on insulin secretion and insulin sensitivity.

In addition to the employment of different animal species or strains, such differences in dietary fat and sugar amounts are likely to explain that a variety of metabolic profiles are developed by experimental animal models in different studies.

Major hypothalamic injury has been proposed to occur within a few days of HFD feeding, this preceding weight gain (Thaler et al., 2012). Thus, early hypothalamic alterations are important determinants for the loss of whole-body metabolic control upon exposure to obesogenic diets. Indeed, regulation of energy balance relies on glucose sensing by neuronal networks that control food intake, hepatic glucose production, and pancreatic counter-regulatory hormone secretion. The hypothalamus is a primary site for integration of peripheral and central neuronal signals and hormonal inputs (Jordan et al., 2010). Impaired hypothalamic glucose sensing is key in developing T2D in obese humans and animal models (Colombani et al., 2009; Thaler et al., 2012; Gaspar et al., 2018).

Despite differences in metabolic phenotypes, all these diabetogenic diet interventions generate metabolic syndrome phenotypes that impact brain function, particularly the performance in hippocampal-dependent memory tasks (Soares et al., 2013; Beilharz et al., 2014; Hsu et al., 2015; Lemos et al., 2016; de Souza et al., 2019; Lizarbe et al., 2019b). For example, rats exposed to one week of high-fat and fructose diet displayed impaired hippocampal insulin signaling, and smaller hippocampal size with synaptic degeneration, reduced neuronal processes, and astrogliosis (Calvo-Ochoa et al., 2014). Rats under a similar diet for 5 days displayed impaired performance in place but not object recognition tasks (Beilharz et al., 2014), which are dependent on hippocampus and perirhinal cortex function, respectively. HFD alone also impairs hippocampal-dependent memory (McNay et al., 2010; Pistell et al., 2010; Lizarbe et al., 2019b). Rodents that were allowed to drink a 35% sucrose solution for 2–3 months while fed a low fat diet also develop hippocampal-dependent spatial memory impairment (Soares et al., 2013; Lemos et al., 2016).

While memory assessments have been mostly focused on spatial memory that depends on hippocampal functioning, other functional domains remain to the thoroughly investigated.

## Hyperglycemia and Brain Glucose Toxicity

It is well established that glucose neurotoxicity upon uncontrolled hyperglycemia contributes to cellular dysfunction through (*i*) increased polyol pathway flux, (*ii*) increased advanced glycation end-product formation, (*iii*) activation of protein kinase C (PKC) isoforms, and (*iv*) increased hexosamine pathway flux (Brownlee, 2001). Since the brain has about fivefold less glucose than plasma (Gruetter et al., 1998; Choi et al., 2001; Duarte et al., 2009b), endothelial cells in cerebral vessels are more susceptible to damage by hyperglycemia than cells in the brain parenchyma. Deterioration of the cerebral vasculature can lead to impaired BBB permeability in diabetes, as well as in aging and neurodegenerative disorders (Ueno et al., 2016). However, there is controversial evidence regarding cerebral microcirculation pathology and BBB dysfunction in rodent models of diabetes or *in vitro* models of chronic hyperglycemia (Andaloussi et al., 2018; Rom et al., 2019, and the references therein).

Measurements of brain-to-plasma glucose concentrations *in vivo* have not confirmed a substantial degree of BBB leakage in streptozotocin-induced diabetic rats maintained under hyperglycemia (>20 mmol/L) for 1 month (Duarte et al., 2009a; Wang et al., 2012), or in insulin resistant GK rats that show sustained fed glycemia of 9–16 mmol/L (Duarte et al., 2019; Girault et al., 2019). Accordingly, Andaloussi et al. (2018) have not observed BBB permeability alterations or morphological changes in brain vasculature of Ins2^*AKITA*^ mice that display sustained hyperglycemia above 20 mmol/L. Nevertheless, gene expression profiles in brain microvessels isolated from models of diabetes point toward deregulated expression of genes related to angiogenesis, inflammation, vasoconstriction and vasodilation, and platelet activation pathways (Rom et al., 2019). Proteomic analyses suggest impaired metabolic activity in microvessels from the cerebral cortex of HFD-exposed mice compared to controls (Ouyang et al., 2014), even though HDF exposure results in limited increases of blood glucose levels (Soares et al., 2018; Lizarbe et al., 2019b). Such alterations are likely to impact brain perfusion and to limit nutrient delivery for fueling neuronal energetics (Glaser et al., 2012; Bangen et al., 2018). In mice, exposure to HFD impairs vascular reactivity (relaxation and contractile responses) and cerebral blood flow of the middle cerebral artery and of intraparenchymal micro vessels in prefrontal cortex and hippocampus, without changes of baseline perfusion (Pétrault et al., 2019). Accordingly, HFD feeding also exacerbates memory impairment induced by carotid occlusion without changes in basal cerebral blood flow (Zuloaga et al., 2016).

In sum, BBB breakdown mechanisms in diabetogenic diets are unlikely to be directly linked to hyperglycemia, but may include alterations of endothelial functions.

## Brain Insulin Resistance

Various metabolic hormones (ghrelin, insulin, leptin, glucagon-like peptide 1), which are key in central regulation of appetite through activation of receptors expressed in brain regions such as the hypothalamus, also play a role in learning and memory (Suarez et al., 2019). Insulin has been considered of particular importance for dementia and early changes of glucose metabolism (Duarte, 2015; Gaspar et al., 2016; Lee et al., 2018). However, it has been debated whether brain insulin resistance and metabolic changes are cause or consequence of neurodegeneration (Stanley et al., 2016; Mullins et al., 2017).

Insulin resistance (when cells do not respond to insulin) occurs in T2D, is associated to increased dementia risk, partly due to poor insulin signaling in neurons (Duarte, 2015). Brains from subjects with dementia and AD downregulated insulin receptors (IRs) and pointed toward a major role of neuronal insulin signaling in AD (Duarte, 2015; Barone et al., 2016; Sharma et al., 2019). Glucose utilization by the brain declines with age and is notably impaired in subjects with early AD, which may be related to insulin action in key areas for memory/cognition (Lee et al., 2018). Interestingly, insulin resistance may be differentially associated with either glucose hypo- or hyper-metabolism across different brain areas (Willette et al., 2015b). In fact, Willette et al. found that peripheral insulin resistance is correlated with reduced glucose metabolism in the brain of AD patients, while a positive correlation was observed in the brain of individuals with MCI that then progress to develop AD. Work on animal models of AD, T2D, or insulin resistance also points toward an association between insulin signaling and AD-like pathology (Duarte, 2015; Triani et al., 2018; Sharma et al., 2019). Diverse clinical trials testing the efficacy of insulin to treat AD and MCI are being conducted (Craft et al., 2012; Chapman et al., 2018; Lee et al., 2018).

Insulin is of particular importance in some specific brain areas: the hypothalamus that centrally regulates body energy homeostasis, the fusiform gyrus that plays a role in object recognition tasks, prefrontal areas that process sensory information, and the hippocampus that is key for memory formation (Heni et al., 2015). Binding of insulin to the IR activates the IR substrates IRS1 and IRS2, which in turn activate signaling cascades for brain function regulation, including metabolic processes in the different brain cells (Mullins et al., 2017). Importantly, insulin regulates the expression of genes necessary for memory consolidation, namely, via the mitogen-activated protein kinase (MAPK) pathway (Kelly et al., 2003; Dou et al., 2005). Insulin also participates in controlling the main cellular metabolic sensor AMP-activated protein kinase (AMPK) (Hardie, 2004; Marinangeli et al., 2016), which might provide a means to afford neuroprotection through metabolic control (Marinangeli et al., 2016). Thus, impaired insulin signaling might contribute to poor fueling of brain activity.

**FIGURE 1:**
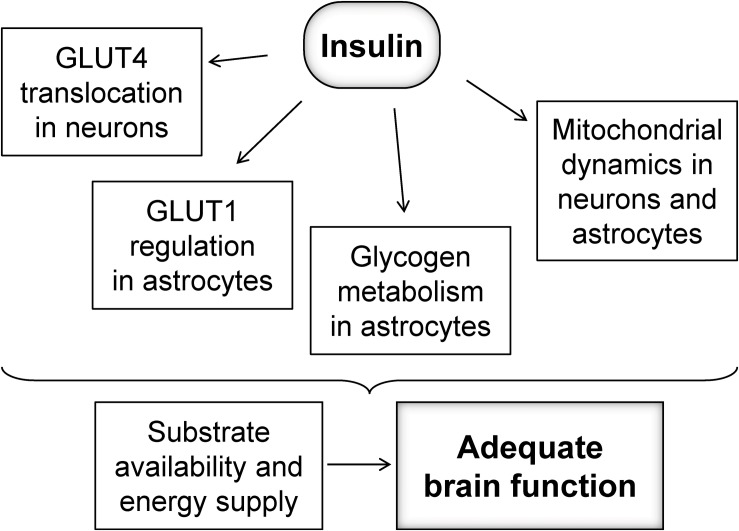
Possible mechanisms by which insulin might regulate fueling of neurons to sustain adequate brain function.

Brain glucose uptake is not dependent on insulin, but might be under control of insulin in specific subcellular compartments (Figure 1). For example, activity at synapses was shown to trigger the mobilization of GLUT4 (the insulin-sensitive glucose carrier) from intracellular sources into axonal plasma membranes, a process that is mediated by the metabolic sensor AMPK, and is necessary for increasing glucose flux into neurons during periods of high metabolic demand, such as during learning (Pearson-Leary and McNay, 2016; Ashrafi et al., 2017). Interestingly, it has been shown that toxic Aβ oligomers impair insulin signaling and decrease plasma membrane translocation of the insulin-sensitive GLUT4 in the hippocampus (Pearson-Leary and McNay, 2012), which might result in poor support of energetic demands within active synapses.

While the focus of insulin signaling has been mostly on neurons, astrocytes also have receptors for insulin and IGF1 that may be key for maintaining GLUT1 at the plasma membrane (Fernandez et al., 2017), and thus regulating glucose utilization. In addition, glycogen metabolism, which is crucial for fueling glutamatergic neurotransmission and memory (Alberini et al., 2018), has also been proposed to be under insulin and IGF-1 regulation in cultured astrocytes (Muhič et al., 2015). Although insulin-dependent glycogen metabolism regulation *in vivo* remains to be elucidated, brain glycogen is mobilized rapidly for supporting glutamatergic neurotransmission *in vivo* (Gibbs et al., 2007), or metabolism during reduced fuel supply (Swanson et al., 1989; Choi et al., 2003; Duarte et al., 2017).

Mitochondria are the power-house of the cell, and mitochondrial dysfunction has been shown to be involved in neurodegenerative processes (Belenguer et al., 2019). Insulin might also control oxidative metabolism in mitochondria of neurons and astrocytes by regulating mitochondrial dynamics, biogenesis or autophagy, oxidative stress and apoptosis (Santos et al., 2014; Westermeier et al., 2015; Ruegsegger et al., 2019). The network of mitochondria is regulated by a fine balance of fission, involving the GTPase dynamin-like protein 1 (DRP1), and fusion processes involving Mitofusin 1 (Mfn1), Mitofusin 2 (Mfn2), and optic atrophy 1 (OPA1) protein (Belenguer et al., 2019). Mitochondrial dynamics dysregulation plays a role in hypothalamic dysfunction upon HFD exposure (Dietrich et al., 2013), and a diabetes-induced increase of DRP1 phosphorylation was observed in the cerebral cortex (Santos et al., 2014). Recently, Ruegsegger et al. (2019) observed increased DRP1 as well as its phosphorylation without changes in Mfn1/2 and OPA1, as well as the expected mitochondrial fragmentation in the HFD-exposed hippocampus. Smaller mitochondria have been associated to reduced oxidative phosphorylation and ATP production rates (Schmitt et al., 2018; Belenguer et al., 2019). Therefore, the loss of mitochondrial metabolism regulation by insulin might result in impaired fueling of neuronal and astrocytic functions.

## Synaptic Dysfunction

Damage of synapses is the most important step for brain dysfunction (Morrison and Baxter, 2012), and the degree of synaptic changes correlates with the severity of cognitive decline (Sheng et al., 2012). Synaptic dysfunction and neurotoxicity in age-associated dementia and AD are mainly caused by amyloid plaques, but also neuroinflammation with reactive microglia (Moore et al., 2019). Mitochondria are also involved in synaptic degeneration due to compromised ATP synthesis (energy failure), as well as impaired Ca^2+^ handling, increased production of reactive oxygen species (ROS), impaired production of metabolites that are neurotransmitter precursors, and dysregulation of mitochondrial dynamics and mitochondria-dependent cell signaling transduction (Tait and Green, 2012; Guo et al., 2017; Belenguer et al., 2019).

Cognitive dysfunction connected to diabetes is particularly associated with significant changes in the integrity of the hippocampus, a brain region considered to mediate memory formation in animals, and electrophysiological analyses indicate that diabetes impairs synaptic plasticity in hippocampal slices (Biessels et al., 2002; Trudeau et al., 2004; Duarte et al., 2019). Based on this, the vast majority of translational studies in animal models of diabetes were dedicated to the study of hippocampal structure and function. Impaired hippocampal-dependent spatial learning and memory have been demonstrated in different animal models of diabetes (e.g., Flood et al., 1990; Duarte et al., 2012a, 2019; Lizarbe et al., 2019b).

Diabetic conditions, including short- and long-term exposure to diets rich in fat and/or sugar, lead to synaptic deterioration that results in defective neurotransmission and synaptic plasticity in the hippocampus (Nitta et al., 2002; Duarte et al., 2009a, 2012a, 2019; Calvo-Ochoa et al., 2014; Girault et al., 2019; Lizarbe et al., 2019b). Interestingly, intranasal insulin treatment in insulin-deficient mice was shown to ameliorate synaptic degeneration and deficits in learning and memory, without preventing hyperglycemia (Francis et al., 2008). This indicates that impairment of central insulin signaling is indeed an important factor for diabetes-induced brain injury.

## Inflammation and Gliosis

The neurodegenerative process in the hippocampus of diabetes models is accompanied by neuroinflammation and astrogliosis (e.g., Saravia et al., 2002; Baydas et al., 2003; Duarte et al., 2009a, 2012a, 2019; Calvo-Ochoa et al., 2014). Inflammation and activation of microglia have been observed in animal models fed diabetogenic diets and have been linked to memory impairment. However, the activation of microglia as consequence of diabetogenic diet exposure has not been consistently observed.

Seven days of feeding a diet rich in fat and fructose induced hippocampal dendritic damage, accompanied by an increase of reactive astrocytes associated with microglial changes (Calvo-Ochoa et al., 2014). Long-term HFD consumption (4 months) increased expression of pro-inflammatory cytokines in hippocampus of rats, namely, IL-6, IL-1β, and TNFα (Dutheil et al., 2016). In contrast, astrogliosis (elevated levels of GFAP) and microgliosis (elevated levels of Iba1) were not observed in the hippocampus of mice exposed to HFD for 6 months (Lizarbe et al., 2019b). Denver et al. (2018) showed astrogliosis and microgliosis in cortex and dentate gyrus of mice fed a HFD for 18 days, but not after 1 month, even though the expression of inflammatory genes such as IKKβ, ERK2, mTOR, NF-kB1, and TLR4 persisted upregulated for 5 months on HFD (Denver et al., 2018). It should be noted, however, that levels of GFAP or Iba1 alone might not report on changes in cellular morphology, and such simplistic assessments might contribute to reported controversies (Gzielo et al., 2017).

Proliferation of microglia might also depend on the age of HFD exposure. Aged animals appear to be more susceptible to develop HFD-induced neuroinflammation (Spencer et al., 2019). Hsu et al. (2015) also observed age-dependent inflammation effects of exposure to sugar-rich diets. Namely, diabetogenic diets rich in sucrose or fructose for 1 month, which that do not result in obesity, triggered memory impairment with some degree of neuroinflammation in the hippocampus of adolescent rats, but not in adults (Hsu et al., 2015).

In sum, neuroinflammation profiles not only change with the duration of HFD exposure, but also depend on the age of onset.

## Brain Energy Metabolism in Diet-Induced T2D

Brain function requires continuous supply of glucose and oxygen and a tight regulation of metabolic interactions between neurons and astrocytes (Sonnay et al., 2017). Loss of this metabolic regulation that fuels neuronal activity has been proposed to be the culprit of memory dysfunction (Alberini et al., 2018), followed by an important neurodegenerative process (de la Monte, 2017).

The predominant glucose carrier isoforms involved in cerebral glucose utilization are GLUT1 and GLUT3. GLUT1 is expressed in all brain cells including the endothelial cells and with very low neuronal expression, while GLUT3 is almost restricted to neurons (Simpson et al., 2007). Levels of the main BBB carrier GLUT1 were found reduced in the hippocampus of insulin resistant GK rats (Soares et al., 2019). In contrast, Vannucci et al. (1997) reported no changes in the density of GLUT1 or GLUT3, in the brain of db/db mice, relative to wild-type mice. Nevertheless, both studies found T2D-induced reduced cerebral glucose utilization (Vannucci et al., 1997; Soares et al., 2019). Lower levels of both GLUT1 and GLUT3 were found in the brain of mice under a diet rich in fat and sugar for 3 months (Kothari et al., 2017). Mice fed an HFD for 3 months also showed reduced density of the neuronal GLUT3, and of the insulin-dependent GLUT4 that is key for synaptic fueling (see above), when compared to controls (Liu et al., 2015). Altogether, this suggests that brain cells, and especially neurons, have reduced access to glucose in the insulin resistant brain.

PET scans using FDG are commonly used to evaluate brain glucose uptake in both humans and animal models, as well as the CMR_*glc*_ utilization. Although insulin is the main regulator of peripheral glucose metabolism, it is considered to not control glucose uptake and utilization in the healthy brain (e.g., Hasselbalch et al., 1999). In contrast, insulin was shown to stimulate brain glucose metabolism in subjects with impaired glucose tolerance (Hirvonen et al., 2011) and there have been reports of inverse relations between insulin resistance and CMR_*glc*_ (Baker et al., 2011; Willette et al., 2015b). Using FDG-PET, Liu et al. (2017) observed lower glucose uptake in the brain of mice fed an HFD for about 2 months, relative to controls.

Mitochondria are key in neurodegeneration processes, namely, due to oxidative phosphorylation dysfunction, impaired Ca^2+^ homeostasis and signaling, and oxidative stress (Belenguer et al., 2019). Impaired mitochondria dynamics resulting in mitochondrial fragmentation was observed in the hippocampus of mice exposed to HFD (Ruegsegger et al., 2019), which might result in reduced energy production (Schmitt et al., 2018). Park et al. (2018) showed that mitochondrial activity is affected in mice fed with HFD. More specifically, they suggested enhanced mitochondrial production of H_2_O_2_, impaired O_2_ consumption, and lower Ca^2+^ retention capacity in the hippocampus of HFD-exposed mice compared to controls. Moreover, the hippocampus of mice fed an HFD for 6 months showed deficits in the respiratory chain and oxidative phosphorylation (at the level of complexes I, II, III, and IV), as well as reduced levels of key proteins for mitochondrial health, such as PGC-1α and TFAM (Petrov et al., 2015). Decrease in mitochondrial respiration, membrane potential, and energy levels was also observed in the cerebral cortex and hippocampus of mice exposed to high sucrose (20%) in the drinking water, defects that are associated to reduced levels of key proteins for mitochondrial function, such as ATG7, LAMP1, ND1, and NRF2 (Carvalho et al., 2015). Although not all diet-induced diabetic phenotypes comprise baseline fed hyperglycemia, increased glucose levels in the brain might contribute to mitochondrial defects (Hinder et al., 2012). Unfortunately, studies of mitochondria from diabetes models have not been designed to distinguish between the different cellular compartments, that is, whether mitochondria originate from neurons or other brain cells. It is plausible that neuronal mitochondria, especially those locate within or near synapses, are key in the process of synaptic deterioration. On the other hand, altered metabolism within processes of reactive astrocytes is likely to contribute for poor support of neurons and synapses.

### Neuron–Astrocyte Metabolic Interactions

There is abundant knowledge on the plethora of molecular events in neurons that define synaptic activity and the electrophysiological basis of memory. By contrast, mechanisms by which other brain cells regulate synaptic functions are less understood. Astrocytes are brain cells that surround synapses, and are well equipped to modulate neuronal functions, namely, those involved in memory formation: they are excitable through Ca^2+^ fluctuations when responding to neurotransmitters released at synapses, synchronize to nearby astrocytes by Ca^2+^ waves, release gliotransmitters that influence synaptic plasticity, communicate to blood vessels thus coupling neuronal activity to nutrient supply from circulation, and regulate energy metabolism in support of neurons (Sonnay et al., 2017). Indeed, there is a tight coupling between oxidative metabolism in astrocytic mitochondria and excitatory glutamatergic neurotransmission, defined by the rate of the glutamate-glutamine cycle (Sonnay et al., 2016, 2018), which is crucial for brain function and memory (Alberini et al., 2018). Notably, astrocytes are also the brain reservoir of glucose storage in the form of glycogen, which is nearly absent in neurons (Duran et al., 2019), and lactate produced by glycogenosis and glycolysis in astrocytes has been proposed to be necessary for fueling brain function and memory (Alberini et al., 2018). While healthy astrocytes support neurons, neuroinflammatory microglia release molecules that favor the formation of a neurotoxic subset of astrocytes called A1. A1 astrocytes lose their normal functions, and also secrete harmful factors that may damage neurons (Liddelow and Barres, 2017). Upon this astrogliosis process, the metabolic support from astrocytes to neurons is likely disrupted.

**FIGURE 2:**
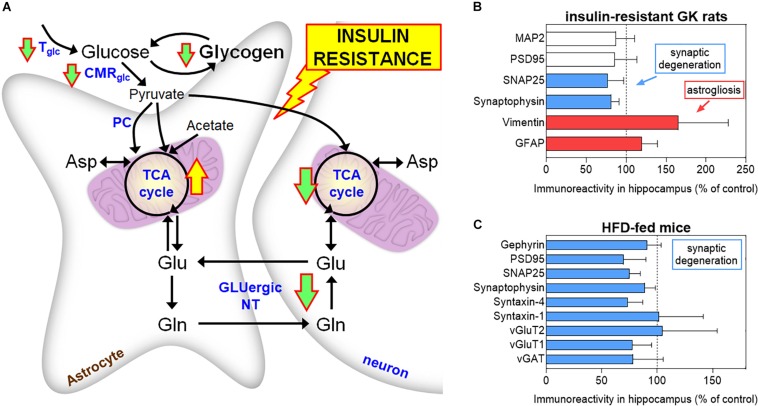
Brain energy metabolism alterations in insulin resistant GK rats **(A)**. Down and up arrows indicate decreased and increased rate of pathways in insulin-resistant GK rats, respectively (Girault et al., 2019; Soares et al., 2019). These alterations are supported by findings of increased astroglial markers (GFAP and vimentin) and reduced levels of synaptic proteins in the hippocampus of GK rats, relative to control Wistar rats **(B)**. Mice exposed to 60% HFD for 6 months also show synaptic degeneration in the hippocampus, as suggested by reduced levels of synaptic proteins, versus 10% fat-fed control mice **(C)**. MAP2 is a neuronal marker, PSD95 and gephyrin are post-synaptic density markers, SNAP25, synaptophysin, and syntaxin-1/4 are located in the presynaptic button, and vGluT1/2 and vGAT are transporters in synaptic vesicles. Data in graphs of **B** and **C** are from Duarte et al. (2019) and Lizarbe et al. (2019a), respectively, and are shown as% of controls (mean ± SD). T_glc_, glucose transport; CMR_glc_, cerebral metabolic rate of glucose; PC, pyruvate carboxylase; TCA, tricarboxylic acid; GLUergic NT, glutamatergic neurotransmission.

In non-obese, insulin-resistant GK rats, T2D is associated to impaired glucose utilization and glutamatergic neurotransmission in neurons, while astrocytes *in vivo* display exacerbated oxidative metabolism and impaired glutamine synthesis (Girault et al., 2019). According to increased mitochondrial metabolism in astrocytes, Liu et al. (2017) reported higher labeling incorporation from [^13^C]acetate (an astrocyte-specific metabolic tracer) into glutamine in HFD-fed mice than controls, without substantial changes of labeling from [^13^C]glucose. Astrocytic glutamine production is particularly importance for excitatory glutamatergic neurotransmission. Furthermore, work on GK rats shows that insulin resistance is associated with defects of astrocytic glycogen metabolism, namely, in the hippocampus that controls learning/memory (Soares et al., 2019). These observations suggest that energy metabolism in astrocytes is dysregulated in diabetes and might contribute to synaptic dysfunction (Figure 2). Such energy metabolism changes result in modified brain metabolic profiles in GK rats, as measured *in vivo* by ^1^H magnetic resonance spectroscopy (MRS), and are accompanied by astrogliosis, loss of synaptic proteins required for neurotransmission, and impaired synaptic plasticity (Duarte et al., 2019). Similar decreased density of proteins that depict synaptic degeneration was verified in the hippocampus of mice fed a HFD for 6 months (Lizarbe et al., 2019b), and obese NONcNZO10/LtJ mice (Duarte et al., 2012a).

### Brain Metabolic Profiles

Modifications of brain metabolism in diet-induced obesity and diabetes are likely to be reflected in brain metabolic profiles, which can be measured *in vivo* by MRS. In diabetes patients, studies have generally observed a reduction in the levels of the putative neuronal marker *N*-acetylaspartate (NAA), as well as an increase in *myo*-inositol content (Duarte, 2016). Levels of *myo*-inositol in brain MRS are considered to reflect the size of the astrocytic metabolic pool (discussed in Duarte et al., 2012c). Alterations in the concentration of these two brain metabolites are generally patent in neurodegenerative disorders, namely, AD, Parkinson’s disease, and Huntington’s disease (Duarte et al., 2014). Moreover, concentrations of both NAA and *myo*-inositol were found to be associated with insulin sensitivity (Karczewska-Kupczewska et al., 2013).

Higher concentrations of *myo-*inositol were also observed in hypothalamus but not hippocampus or cortex of mice fed a 60% HFD during 6 months (Lizarbe et al., 2019a, b). However, rather than reduced NAA levels, these MRS experiments have found an increase of NAA content particularly prominent in the hippocampus. This NAA increase may be linked to changes of osmolarity since the concentration of other major osmolites such as taurine and creatine was also observed. In rats under 60% HFD for 5 months, Raider et al. (2016) have observed no changes in hippocampal NAA but reduced levels of *myo*-inositol, compared to controls. Hassan et al. (2018) also observed HFD-induced metabolic changes in extracts from the prefrontal cortex, namely, higher relative concentrations of lactate, alanine, taurine, and *myo*-inositol, and lower GABA levels. Some metabolic alterations were also observed in the mouse striatum, but not in the hippocampus and hypothalamus. Differences between metabolic profiles *in vivo* and *post mortem* might contribute to the differences in these studies. However, further work must be undertaken to understand the cause of metabolic profile changes in the hippocampus of mice under diabetogenic diets.

## Conclusion

Diet-induced metabolic syndrome or T2D in rodents show variable phenotypes depending on the employed diet. Nevertheless, all models show robust effects on memory performance, particularly in spatial tasks that rely on adequate hippocampal function. Across the available literature, one observes that metabolism alterations underlying memory impairment include alterations of glucose utilization in neurons and astrocytes, dysfunctional mitochondria in neurons but exacerbated oxidative metabolism in astrocytes, which is likely required to sustain T2D-induced astrocyte hyper-reactivity. Despite increased astroglial metabolism, the metabolic support from astrocytes to neurons is not adequate, and might contribute to synaptic dysfunction and memory derangements. The mechanisms by which insulin differentially regulates metabolism in neurons and astrocytes require further investigation, in order to understand brain insulin resistant development and how it leads to impaired cognition.

The interaction of insulin with other neuromodulation systems that regulate cell signaling and metabolism has been proposed but insufficiently investigated. For example, IRs interact with the endocannabinoid system (Dalton and Howlett, 2012; Kim et al., 2012) that modulates neuronal and astrocytic metabolism (Duarte et al., 2012b; Köfalvi et al., 2016), and with biliverdin reductase-A that modulates cellular stress responses (Barone et al., 2016). Such signaling interactions may be key for insulin to fulfill its glucose uptake-unrelated roles, and may reveal to be therapeutic targets against brain dysfunction.

Finally, while aging is a key factor on the development of insulin resistance, there is a major knowledge gap on the T2D-aging interaction leading to dysregulation of cerebral metabolism. Suggestions of the time complexity of brain insulin resistance mechanisms come from longitudinal studies in humans. For example, it is known that mid-life obesity is associated with an increased risk of incident dementia (see above), but late-life obesity was found to be negatively associated with incident dementia (Pedditzi et al., 2016). Moreover, insulin resistance was proposed to be associated with glucose cerebral hypo-metabolism in AD patients, but associated to hyper-metabolism in subjects with MCI that will later progress to AD (Willette et al., 2015b). Further research is needed to identify trajectories of insulin-dependent brain metabolism dysregulation leading to brain dysfunction.

## Author Contributions

Both authors wrote the manuscript.

## Conflict of Interest

The authors declare that the research was conducted in the absence of any commercial or financial relationships that could be construed as a potential conflict of interest.

## Abbreviations

A β: amyloid β
AD: Alzheimer’s disease
BBB: blood–brain barrier
CMR_*glc*_: cerebral metabolic rate of glucose
FDG: [^18^F]-fluorodeoxyglucose
GK: Goto-Kakizaki
HFD: high-fat diet
IGF1: insulin-like growth factor 1
IR: insulin receptor
MCI: mild cognitive impairment
MRS: magnetic resonance spectroscopy
NAA: *N*-acetylaspartate
PET: positron emission tomography
T2D: type 2 diabetes.
